# Current practical experience with artificial intelligence in clinical radiology: a survey of the European Society of Radiology

**DOI:** 10.1186/s13244-022-01247-y

**Published:** 2022-06-21

**Authors:** European Society of Radiology (ESR), Christoph D. Becker, Elmar Kotter, Laure Fournier, Luis Martí-Bonmatí

**Affiliations:** European Society of Radiology (ESR), Am Gestade 1, 1010 Vienna, Austria

**Keywords:** Professional issues, Artificial intelligence in imaging, Artificial intelligence and workload, Artificial intelligence in radiology

## Abstract

A survey among the members of European Society of Radiology (ESR) was conducted regarding the current practical clinical experience of radiologists with Artificial Intelligence (AI)-powered tools. 690 radiologists completed the survey. Among these were 276 radiologists from 229 institutions in 32 countries who had practical clinical experience with an AI-based algorithm and formed the basis of this study. The respondents with clinical AI experience included 143 radiologists (52%) from academic institutions, 102 radiologists (37%) from regional hospitals, and 31 radiologists (11%) from private practice. The use case scenarios of the AI algorithm were mainly related to diagnostic interpretation, image post-processing, and prioritisation of workflow. Technical difficulties with integration of AI-based tools into the workflow were experienced by only 49 respondents (17.8%). Of 185 radiologists who used AI-based algorithms for diagnostic purposes, 140 (75.7%) considered the results of the algorithms generally reliable. The use of a diagnostic algorithm was mentioned in the report by 64 respondents (34.6%) and disclosed to patients by 32 (17.3%). Only 42 (22.7%) experienced a significant reduction of their workload, whereas 129 (69.8%) found that there was no such effect. Of 111 respondents who used AI-based algorithms for clinical workflow prioritisation, 26 (23.4%) considered algorithms to be very helpful for reducing the workload of the medical staff whereas the others found them only moderately helpful (62.2%) or not helpful at all (14.4%). Only 92 (13.3%) of the total 690 respondents indicated that they had intentions to acquire AI tools. In summary, although the assistance of AI algorithms was found to be reliable for different use case scenarios, the majority of radiologists experienced no reduction of practical clinical workload.

## Key points

- Artificial Intelligence (AI) algorithms are being used for a large spectrum of use case scenarios in clinical radiology in Europe, including assistance with interpretive tasks, image post-processing, and prioritisation in the workflow.
- Most users considered AI algorithms generally reliable and experienced no major problems with technical integration in their daily practice.
- Only a minority of users experienced a reduction of the workload of the radiological medical staff due to the AI algorithms.

## Background and objectives

Digital imaging is naturally predisposed to benefit from the rapid and exciting progress in data science. The increase of imaging examinations and the associated diagnostic data volume have resulted in a mismatch between the radiologic workforce and workload in many European countries. In an opinion survey conducted in 2018 among the members of the European Society of Radiology (ESR), many respondents had expectations that algorithms based on artificial intelligence (AI) and particularly machine learning could reduce radiologists’ workload [1]. Although a growing number of AI-based algorithms has become available for many radiological use case scenarios, most published studies indicate that only very few of these tools are helpful for reducing radiologists’ workload, whereas the majority rather result in an increased or unchanged workload [2]. Furthermore, in a recent analysis of the literature it was found that the available scientific evidence of the clinical efficacy of 100 commercially available CE-marked products was quite limited, leading to the conclusion that AI in radiology was still in its infancy [3]. The purpose of the present survey was to get an impression of the current practical clinical experience of radiologists from different European countries with AI-powered tools.

## Methods

**Fig. 1:**
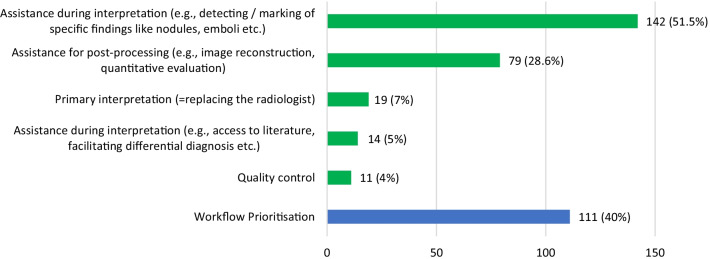
Which type of scenario (use case) was addressed by the used AI algorithm(s) in clinical routine? The answers of all 276 respondents with practical clinical AI experience are shown, including the number of respondents using one or more algorithms for assistance in diagnostic interpretation (green) and/ or workflow prioritisation (blue)

**Fig. 2:**
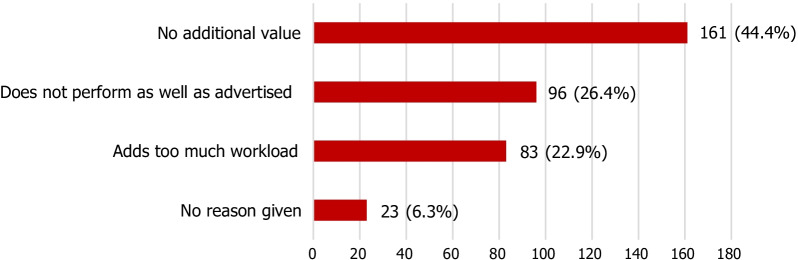
Reasons given by 363 of all 690 participants of the survey (regardless of their experience with AI-based algorithms in clinical workflow) for not intending to acquire a certified AI-based algorithm for their clinical practice

A survey was created by the members of the ESR eHealth and Informatics Subcommittee and was intentionally kept brief to allow responding in a few minutes. A few demographic questions included the country, type of institution (i.e. academic department, regional hospital, or private practice), and the main field of radiological practice as summarised in Tables 1, 2 and 3. For the more specific questions about the use of AI-based algorithms it was clearly stated that the answers were intended to reflect experience from clinical routine rather than research and testing purposes. The questions related to the use of AI addressed the respondents’ working experience with certified AI-based algorithms, possible difficulties in integrating these algorithms in the IT system, and different use case scenarios for which AI-based algorithms were used in clinical routine, mainly distinguishing between tools aiming at facilitating the diagnostic interpretation process itself (questions shown in Fig. 1) from those that were aiming at facilitating the prioritisation of examinations in the workflow. Specific questions addressed the technical integration of the algorithms (question mentioned in Table 4); radiologists’ confidence in the diagnostic performance (question mentioned in Table 5); quality control mechanisms to evaluate diagnostic accuracy (questions mentioned in Tables 6, 7 and 8); communication of the use of diagnosis-related algorithms towards patients or in the radiology reports (questions mentioned in Tables 9 and 10); and the usefulness of algorithms for reducing the radiologists’ workload (questions mentioned in Tables 11 and 12). Respondents also had the opportunity to offer free text remarks regarding their use of AI-based tools. Those respondents who did not use AI-based algorithms for the purpose of clinical practice were asked to skip all the questions related to clinical AI-use and to proceed directly to the last question about acquisition of AI-based algorithms, so that the opinions of all participating radiologists were taken into consideration for the final questions about their intentions regarding acquisition of such tools (question mentioned in Fig. 2).

**Table 1:** Distribution of all 690 respondents by countries and proportion of radiologists with practical clinical experience with AI algorithms

Country	Number of respondents per country	Number of respondents with practical clinical experience with AI per country	Percentage of radiologists with practical clinical experience in AI per country (%)
Italy	71	23	32
Spain	64	19	30
UK	60	23	38
Germany	50	23	46
Netherlands	50	35	70
Sweden	29	14	48
Denmark	27	15	56
Turkey	27	3	11
Norway	26	12	46
Switzerland	27	14	54
France	25	12	48
Belgium	23	13	57
Austria	21	12	57
Greece	21	5	24
Portugal	17	5	29
Romania	16	4	25
Ukraine	13	3	23
Croatia	11	4	36
Russian Fed	11	4	36
Bulgaria	10	0	0
Poland	10	4	40
Finland	7	4	57
Hungary	7	3	43
Serbia	7	1	14
Slovenia	7	3	43
Slovakia	6	5	83
Ireland	5	2	40
Lithuania	5	2	40
Bos. & Herzegovina	4	0	0
Czech Republic	4	3	75
Israel	4	2	50
Latvia	4	0	0
Armenia	3	0	0
Albania	2	0	0
Azerbaijan	2	0	0
Belarus	2	0	0
Estonia	2	2	100
Georgia	2	0	0
Kazakhstan	2	0	0
Luxembourg	2	1	50
Cyprus	1	0	0
Iceland	1	0	0
Kosovo	1	1	100
Uzbekistan	1	0	0
Total	690	276

**Table 2:** Respondents with practical clinical experience with AI-based algorithms: distribution of origin by countries and type of institutions

Country	Number of respondents per country	Number of institutions per country	Respondents from academic departments	Respondents from private practice	Respondents from regional hospitals
Netherlands	35	20	16	0	19
Germany	23	21	14	3	6
Italy	23	21	13	0	10
UK	23	22	7	2	14
Spain	19	16	14	1	4
Denmark	15	7	11	1	3
Switzerland	14	13	6	6	2
Sweden	14	14	7	1	6
Belgium	13	9	5	1	7
Austria	12	11	7	1	4
France	12	11	5	5	2
Norway	12	9	6	0	6
Greece	5	5	2	2	1
Portugal	5	4	0	4	1
Slovakia	5	5	2	2	1
Croatia	4	4	1	1	2
Finland	4	3	3	0	1
Poland	4	3	3	0	1
Romania	4	2	2	0	2
Russian Fed	4	4	3	0	1
Czech Republic	3	3	1	0	2
Hungary	3	3	2	0	1
Slovenia	3	3	2	0	1
Turkey	3	3	3	0	0
Ukraine	3	2	2	1	0
Estonia	2	2	1	0	1
Ireland	2	2	1	0	1
Israel	2	2	2	0	0
Lithuania	2	2	0	0	2
Kosovo	1	1	1	0	0
Luxembourg	1	1	0	0	1
Serbia	1	1	1	0	0
Total	276	229	143 (52%)	31 (11%)	102 (37%)

**Table 3:** Respondents with practical clinical experience with AI-based algorithms: main field of activity/subspecialty

Field of practice	Number of respondents	(%)
Abdominal radiology	45	16.3
Neuroradiology	45	16.3
General radiology	39	14.1
Chest radiology	32	11.6
Cardiovascular radiology	24	8.7
Musculoskeletal radiology	23	8.3
Oncologic imaging	23	8.3
Breast radiology	17	6.2
Emergency radiology	10	3.6
Paediatric radiology	8	2.9
Urogenital radiology	6	2.2
Head and Neck radiology	4	1.5
Total	276	100

**Table 4:** Respondents with practical clinical experience with AI-based algorithms: Have there been any major problems with integration of AI-based algorithms into your IT system/workflow?

Answer	Number of respondents	(%)
Yes	49	17.8
No	123	44.5
Skipped	104	37.7
Total	276	100

**Table 5:** Experience of 185 respondents with AI-based algorithms for clinical diagnostic interpretive tasks: Were the findings of the algorithm(s) considered to be reliable?

Answer	Number of respondents	(%)
Yes	140	75.7
No	31	16.8
Skipped	14	7.5
Total	185	100

**Table 6:** Experience of 185 respondents with AI-based algorithms for clinical diagnostic interpretive tasks: Were discrepancies between the software and the radiologist recorded?

Answer	Number of respondents	(%)
Yes	82	44.4
No	89	48.1
Skipped	14	7.5
Total	185	100

**Table 7:** Experience of 185 respondents with AI-based algorithms for clinical diagnostic interpretive tasks: Was the diagnostic accuracy (ROC curves) supervised on a regular basis in comparison with the radiologist's diagnosis?

Answer	Number of respondents	(%)
Yes	63	34.1
No	108	58.4
Skipped	14	7.5
Total	185	100

**Table 8:** Experience of 185 respondents with AI-based algorithms for clinical diagnostic interpretive tasks: Was the diagnostic accuracy (ROC curves) supervised on a regular basis in comparison with the final diagnosis in the medical record?

Answer	Number of respondents	(%)
Yes	56	30.3
No	115	62.2
Skipped	14	7.5
Total	185	100

**Table 9:** Experience of 185 respondents with AI-based algorithms for clinical diagnostic interpretive tasks: Were patients informed that an AI software was used to reach the diagnosis?

Answer	Number of respondents	(%)
Yes	32	17.3
No	139	75.2
Skipped	14	7.5
Total	185	100

**Table 10:** Experience of 185 respondents with AI-based algorithms for clinical diagnostic interpretive tasks: Was the use of an AI software to reach the diagnosis mentioned in the report?

Answer	Number of respondents	(%)
Yes	64	34.6
No	107	57.9
Skipped	14	7.5
Total	185	100

**Table 11:** Experience of 185 respondents with AI-based algorithms for clinical diagnostic interpretive tasks: Has (have) the algorithm(s) used for diagnostic assistance proven to be helpful in reducing the workload for the medical staff?

Answer	Number of respondents	(%)
Yes	42	22.7
No	129	69.8
Skipped	14	7.5
Total	185	100

**Table 12:** Experience of 111 respondents with AI-based algorithms for clinical workflow prioritisation: Has the algorithm proven to be helpful in reducing the workload for the medical staff?

Answer	Number of respondents	(%)
Not at all helpful	16	14.4
Moderately helpful	69	62.2
Very helpful	26	23.4
Total	111	100

The survey was created through the ESR central office using the “Survey Monkey platform” (SurveyMonkey Inc., San Mateo, CA, USA) and 27,700 radiologist members of the ESR were invited by e-mail to participate in January 2022. The survey was closed after a second reminder in March 2022. The answers of the respondents were collected and analysed using an Excel software (Microsoft, Redmond, WA, USA).

## Results

A total of 690 ESR radiologist members from 44 countries responded to the survey, for a response rate of 2.5%. The distribution per country and the proportion of respondents with practical clinical experience with AI-based algorithms per country are given in Table 1.

The 276 respondents with practical clinical experience with AI-based algorithms were affiliated to 229 institutions in 32 countries; their answers formed the main basis of this study. Table 2 shows that 143 (52%) of the respondents with practical clinical experience with AI algorithms were affiliated to academic institutions, whereas 102 (37%) worked in regional hospitals, and 31 (11%) in private practice.

Table 3 characterises the same group of respondents as in Table 2 regarding their main field of activity showing that a wide range of subspecialties was represented in the survey and that abdominal radiology, neuroradiology, general radiology, and emergency radiology together accounted for half of the respondents. A detailed analysis of the results according to subspecialties was beyond the scope of the study because of the relatively small number of resulting groups.

The experience regarding technical integration of the software algorithms into the IT system or workflow is summarised in Table 4, showing that only 17.8% of respondents reported difficulties with integration of these tools, whereas a majority of 44.5% observed no such difficulties, although 37.7% of respondents did not answer this question.

Algorithms were used in clinical practice either for assistance in interpretation or for prioritisation of workflow. An overview of the scenarios for which AI- powered algorithms were the used by the respondents is given in Fig. 1.

### Use of algorithms for assistance in diagnostic interpretation

Among the 276 respondents who shared their practical experience with AI-based tool experience, a total of 185 (67%) reported clinical experience with one or more integrated algorithms for routine diagnostic tasks. As seen in Fig. 1 there were different use case scenarios, the commonest being detection or marking of specific findings. The free text remarks of the respondents showed a large range of pathologies in practically all clinical fields and with almost all imaging modalities. Typical examples of pathologies were pulmonary emboli and parenchymal nodules, cerebral haemorrhage and reduced cerebrovascular blood flow, or colonic polyps on CT. Other tasks included the detection of traumatic lesions, e.g. the presence of bone fractures on conventional radiographs or the calculation of bone age. The second most common diagnostic scenario was assistance with post-processing (e.g. using AI-based tools for image reconstruction or quantitative evaluation of structural or functional abnormalities), followed by primary interpretation (i.e. potentially replacing the radiologist), assistance with differential diagnosis, e.g. by facilitation of literature search, and quality control.

Although a detailed analysis of all different diagnostic use case scenarios was beyond the scope of this survey, the respondents’ answers to specific survey questions are shown in Tables 5, 6, 7, 8, 9, 10 and 11. Because some respondents skipped or incompletely answered some questions, the number of yes/no answers per question was not complete. As shown in Table 5, most respondents (75.7%) found the results provided by the algorithms generally reliable.

A significant number of respondents declared that they used mechanisms of quality assurance regarding the diagnostic performance of the algorithms. These included keeping records of diagnostic discrepancies between the radiologist and the algorithms in 44.4%, establishing receiver-operator characteristic (ROC) curves of diagnostic accuracy based on the radiologist’s diagnosis (34.1%) and/ or ROC curves based on the final medical record (30.3%) (Tables 6, 7 and 8).

The use of a diagnostic algorithm was disclosed to patients by 17.3% of the respondents but mentioned in the report by 34.6% (Tables 9 and 10).

Only a minority of 22.7% of respondents who used AI-based algorithms for diagnostic purposes experienced a reduction of their workload, whereas 69.8% reported that there was no reduction effect on their workload (Table 11).

### Use of algorithms for prioritisation of workflow

Among the 276 respondents who had practical experience with AI-based tools, there were 111 respondents (40%) reporting experience with algorithms for prioritisation of image sets in their clinical workflow. As shown in Table 12, the prioritisation algorithms were considered to be very helpful for reducing the workload of the medical staff by 23.4% respondents who used them, whereas the other users found them only moderately helpful (62.2%) or not helpful at all (14.4%).

### Intentions of all respondents regarding the acquisition of an AI-based algorithm

All participants of the survey, regardless of their practical clinical experience, were given the opportunity to answer the question whether they intended to acquire a certified AI- based software. Of the 690 participants, 92 (13.3%) answered “yes”, 363 (52.6%) answered “no,” and 235 (34.1%) did not answer this question. Figure 2 summarises the reasons given by participants who did not intend to acquire AI-based algorithms for their clinical use.

## Discussion

While the previous survey on AI [1] was based on the expectations of the ESR members regarding the impact of AI on radiology, the present survey intended to obtain an overview of current practical clinical experience with AI-based algorithms. Although the respondents with practical clinical experience in this survey represent only 1% of the ESR membership, their proportion among all respondents varied greatly among countries. The geographical distribution of the 276 radiologists who shared their experience with such tools in clinical practice shows that the majority was affiliated to institutions in Western and Central Europe or in Scandinavia. Half of all respondents with practical clinical experience with AI tools was affiliated to academic institutions, whereas the other half practiced radiology in regional hospitals or in private services. Since it is likely that the respondents in this survey were radiologists with a special interest in AI-based algorithms, it cannot be assumed that this survey reflects the true proportion of radiologists in the European region with practical clinical experience with AI-based tools.

Most of the respondents of this brief survey did not encounter major problems related to the integration the AI-based software tools into the local IT systems; less than 18% did have such issues. However, it must be taken into consideration that radiologists are not always directly involved in the technical process of software integration; this fact may perhaps also explain the relatively high number of respondents who did not reply to this specific question.

Today, AI-based tools for diagnostic purposes may address a large range of use case scenarios. Although this was reflected by the free text answers of the respondents of the present study, the present survey distinguished mainly between algorithms for diagnostic purposes and those for the prioritisation of workflow whereas a detailed analysis of all the different individual use case scenarios was beyond the scope of this survey. Since diagnostic tools are usually quite specific and related to organs and pathologies, even radiologists working in the same institution but in different subspecialties may have different experiences with different algorithms related to their respective fields.

In a recent survey among the members of the American College of Radiology (ACR) the most common applications for AI were intracranial haemorrhage, pulmonary embolism, and mammographic abnormalities, although it was stated that in the case of mammography, confusion must be avoided between AI-based tools and the more traditional software for computer aided diagnosis (CAD) [4]. It was estimated that AI was used by approximately 30% of radiologists, but concerns over inconsistent performance and a potential decrease in productivity were considered to be barriers limiting the use of such tools. Over 90% of respondents would not trust these tools for autonomous use. It was concluded that despite initial predictions the impact of AI on clinical practice was modest [4].

Quality assurance of algorithms that are based on machine–learning may be quite time-consuming and requires considerable resources. Effective supervision of the sensitivity and specificity of a device that adapts itself over time may be done by recording differences between the diagnosis of the radiologist and the algorithm but ideally combines regular monitoring by comparison against a final diagnosis as a gold standard—a so-called “ground truth”. Despite the enthusiasm about AI-based tools there are some barriers to be addressed when implementing this new technology in clinical practice. These include the large amount of annotated image data required for supervised learning as well as validation and quality assurance for each use case scenario of these algorithms, and, last but not least, regulatory aspects including certification [5, 6]. A recent overview of commercially available CE-marked AI products for radiological use found that scientific evidence of potential efficacy of level 3 or higher was documented in only 18 of 100 products from 54 vendors and that for most of these products evidence of clinical impact was lacking [3].

Nonetheless, as a general impression, most of the respondents of this ESR survey who used AI-based algorithms in their clinical practice considered their diagnostic findings to be reliable for the spectrum of scenarios for which they were used. It is noteworthy that 44% of the respondents recorded discrepancies occurring between the radiologists’ and the algorithms’ findings and that approximately one-third indicated that they generated ROC curves based on the radiological report or the clinical record in order to calculate the performance of algorithms in clinical practice. Details regarding the methodologies, e.g. the degree of automation used for establishing these data, were neither asked from nor provided by the respondents. However, since over one-half of the respondents worked in academic institutions, it is possible that some of the algorithms were not only evaluated in the context of clinical routine but also in the context of scientific research studies, thus explaining the relatively high level of quality supervision of the algorithms. Only a small minority of radiologists participating in this survey informed the patients about the use of AI for the diagnosis and about one-third mentioned it in their reports. This may be understandable as long as the radiologist and not the algorithm makes the final diagnosis.

However, the important question remains to what extent AI-powered tools can reduce radiologists’ workload. In the previous ESR survey conducted in 2018, 51% of respondents expected that the use of AI tools would lead to a reduced reporting workload [1]. The actual contributions of AI to the workload of diagnostic radiologists were assessed in a recent analysis based on large number of published studies. It was concluded that although there was often added value to patient care, workload was decreased in only 4% but increased in 48% and remained unchanged in 46% institutions [2]. The results of the present survey are somewhat more optimistic since almost 23% of respondents experienced a reduction of their workload when using algorithms for diagnostic assistance in clinical practice, whereas almost 70% did not. Observations with algorithms aiming at workflow prioritisation were comparable. In view of the wide range of use case scenarios for which AI- based tools can be applied, additional studies are needed in order to determine for which specific tasks and questions in which subspecialties AI-based algorithms could be helpful to reduce radiologists’ workload. Typically, this could be the case in those scenarios that address the detection of relatively simple diagnostic findings and a high volume of cases.

The previous ESR survey from 2018 included 675 participants of which 20% were already using AI-powered tools and 30% planned to do so [1]. The present ESR survey included 690 participants of which 276 (40%) had experience with such tools in clinical practice. However, when all the participants of the present survey were asked whether they intended to acquire a certified AI-based algorithm, only a minority (13.3%) answered yes, whereas the majority either answered no (52.6%) or did not answer the question (34.1%). Reasons given for the negative answers included doubts about the added value or the advertised performance or concerns regarding added workload. We must consider, however, that the answers to this particular question included not only the opinions of the respondents who had experience with practical clinical use but also of those who used these algorithms rather in the context of scientific projects including non-commercial, home-grown AI-based tools.

The results of the present ESR survey are difficult to compare with the recent ACR survey [4] not only because the questions were not identical, but also because of the existing diversity among European countries. Nonetheless, both surveys conclude that, compared with initial predictions and expectations, the overall impact of AI-based algorithms on current radiological practice is modest.

Several limitations of this brief survey need to be mentioned. Firstly, the survey data cannot reflect the true proportion of European radiologists using AI. Secondly, the answers to several questions can only provide a general overview, although some of the issues addressed by this survey would deserve a more detailed analysis. This is true, for example, regarding the differentiation of use case scenarios as well as the methodologies used for the verification of their results. Thirdly, the observations are based on the situation in 2022, and results and opinions may change rapidly in this evolving field.

In summary, this survey suggests that, compared with initial expectations, the use of AI- powered algorithms in practical clinical radiology today is limited, most importantly because the impact of these tools on the reduction of radiologists’ workload remains unproven. As more experience with AI-powered algorithms for specific scenarios is being gained and some of the barriers to their use may become mitigated in the future, a follow-up to this initial survey could provide further insights into the usefulness of these tools.

## Data Availability

The datasets generated during and/or analysed during the current study are available from the corresponding author on reasonable request.

## Abbreviations

ACR: American College of Radiology
AI: Artificial intelligence
CAD: Computer-aided diagnosis
CE: Conformité Européenne: a self-declaration mark used by manufacturers, intended to prove compliance with EU health and safety regulations
ESR: European Society of Radiology
ROC: Receiver-operator characteristic
